# Discovery of Food-Derived Dipeptidyl Peptidase IV Inhibitory Peptides: A Review

**DOI:** 10.3390/ijms20030463

**Published:** 2019-01-22

**Authors:** Rui Liu, Jianming Cheng, Hao Wu

**Affiliations:** 1 Jiangsu Key Laboratory of Research and Development in Marine Bio-resource Pharmaceutics, Nanjing University of Chinese Medicine, Nanjing 210023, China; liurui@njucm.edu.cn; 2 Jiangsu Collaborative Innovation Center of Chinese Medicinal Resources Industrialization, National and Local Collaborative Engineering Center of Chinese Medicinal Resources Industrialization and Formulae Innovative Medicine, Nanjing 210023, China; 3 School of Pharmacy, Nanjing University of Chinese Medicine, Nanjing 210023, China; 4 School of Pharmacy, University of Wisconsin-Madison, Madison, WI 53705, USA

**Keywords:** dipeptidyl-peptidase IV inhibition, food proteins, peptides, type 2 diabetes mellitus

## Abstract

Diabetes is a chronic metabolic disorder which leads to high blood sugar levels over a prolonged period. Type 2 diabetes mellitus (T2DM) is the most common form of diabetes and results from the body’s ineffective use of insulin. Over ten dipeptidyl peptidase IV (DPP-IV) inhibitory drugs have been developed and marketed around the world in the past decade. However, owing to the reported adverse effects of the synthetic DPP-IV inhibitors, attempts have been made to find DPP-IV inhibitors from natural sources. Food-derived components, such as protein hydrolysates (peptides), have been suggested as potential DPP-IV inhibitors which can help manage blood glucose levels. This review focuses on the methods of discovery of food-derived DPP-IV inhibitory peptides, including fractionation and purification approaches, in silico analysis methods, in vivo studies, and the bioavailability of these food-derived peptides. Moreover, food-derived DPP-IV inhibitory peptides discovered during this decade are listed and distributed in a 3D scatter plot graph based on their IC_50_, molecular weight, and grand average of hydropathicity values, which can help us to understand the relationship between the features of the peptides and their activities.

## 1. Introduction

Diabetes is a chronic metabolic disorder which leads to high blood sugar levels over a prolonged period. Type 2 diabetes mellitus (T2DM), type 1 diabetes mellitus (T1DM), and gestational diabetes mellitus are the most frequent forms; other specific types exist that are much less common [1,2]. In recent years, diabetes has become one of the leading causes of death worldwide. According to the International Diabetes Federation (IDF), it is estimated that about 425 million people were living with diabetes globally in 2017 and that the number will be 642 million by 2040 [3]. T2DM is the most common form of diabetes and results from the body’s ineffective use of insulin: it accounts for the vast majority of people with diabetes around the world. The World Health Organization (WHO) estimates that 90 percent of people living with diabetes have type 2 disease [4]. The exact cause of T2DM remains unclear [5]. The involvement of insulin resistance and pancreatic β-cell dysfunction in the pathogenesis of T2DM has been discussed for a long time [6]. Kahn et al. determined the relationship between insulin sensitivity and β-cell function, and pointed out that the relationship was similar to a feedback loop control system [7]. After insulin-sensitive tissues, such as skeletal muscle, adipose tissue, and liver, take up glucose, these tissues send a feedback signal to the β-cells that they need insulin. In order to maintain normal glucose homoeostasis, β-cells release a certain concentration of insulin. If insulin resistance occurs, the feedback signal from tissues causes the β-cells to increase insulin output to maintain normal glucose tolerance. However, under the situation of insulin resistance, and once the β-cells become unable to release sufficient insulin, glucose concentrations will increase [6].

Genetic and environmental factors are reported to be crucial determinants of insulin resistance and β-cell dysfunction. Genomic investigation has shown that more than 50 gene loci are associated with T2DM [8]. Environmental factors, including energy expenditure, caloric intake, nutrient composition, environmental chemicals, and even the gut environment and gut microbiome, are important as well [9,10]. Aside from those factors, the brain–gut axis also plays an important role in normal glucose homoeostasis. The gastrointestinal tract can release various peptides which are closely related to T2DM, such as glucagon-like peptide 1 (GLP-1) and glucose-dependent insulinotropic polypeptide (GIP). GLP-1 and GIP are well known as incretins and can increase insulin secretion and suppress glucagon secretion [11]. GLP-1 and GIP are released into the small intestine and regulate glucose homoeostasis. The incretin effect, defined as the insulin secretory response of the incretins, accounts for at least 50% of the total insulin secreted after a meal [12]. However, both GLP-1 and GIP are rapidly deactivated by an enzyme: dipeptidyl peptidase IV (DPP-IV). DPP-IV can cleave the N-terminal dipeptide residues of GLP-1 and GIP and produce inactive incretins, which leads to GLP-1 and GIP losing their insulinotropic activity [13,14].

DPP-IV is a homodimeric serine peptidase, which consists of approximately 700 amino acids in each subunit. DPP-IV preferentially cleaves substrate peptides with Pro or Ala at the penultimate position of peptides. DPP-IV is responsible for the degradation of GLP-1 and GIP [15]. DPP-IV inhibition is a key target in the treatment of T2DM, and DPP-IV inhibitors were one of the first classes of oral antidiabetic drugs to be prospectively designed as anti-hyperglycemic agents [16]. To date, more than ten DPP-IV inhibitory drugs, which are classified as gliptins, have been developed and marketed around the world [17]. In 2006 Sitagliptin was the first gliptin to be approved by the United States Food and Drug Administration [18], and new members continue to be approved, such as Vildagliptin, Saxagliptin, Linagliptin, Gemigliptin, Anagliptin, Teneligliptin, Alogliptin, Trelagliptin, Omarigliptin, Evogliptin, and Gosogliptin [16]. However, these synthetic DPP-IV inhibitors are reported to have some adverse effects [19], such as gastrointestinal adverse effects [20], allergic reactions [21], skin-related side effects [22], and musculoskeletal disorders [23].

However, DPP-IV inhibitors have been discovered in foods, herbal preparations, natural sources, and traditional Chinese medicines, including phenolic compounds from blueberry–blackberry wine blends [24], alkaloids from seed extract of *Castanospermum australe* [25], and procyanidins from grape seed [26], all of which have shown good DPP-IV inhibitory activity. Various foods, including milk, fish, wheat gluten, beans, egg, and bivalve mollusks, are natural protein sources; after enzymatic hydrolysis, microbial fermentation, decoction, or some other physical and/or chemical processing, their proteins may be degraded and release various DPP-IV inhibitory peptides [27,28,29,30,31,32]. It has been reported different peptides showed different DPP-IV inhibitory modes, including competitive, uncompetitive, noncompetitive and mixed-type modes, which means those peptides might exert DPP-IV inhibitory activity by binding either at the active site and/or outside the catalytic site of DPP-IV [5]. It has been suggested that those natural food- or herb-derived constituents should be safer than synthetic forms, and could be used for glycemic management. Among the sources of DPP-IV inhibitors, food protein-derived DPP-IV inhibitory peptides have attracted the attention of more and more researchers, owing to their high efficacy and safety [33]. The purpose of this review is to describe the discovery of food-derived DPP-IV inhibitory peptides, and to provide information on their use in glycemic management and blood glucose regulation.

## 2. Methods for Discovering Food-Derived DPP-IV Inhibitory Peptides

### 2.1. Enzymatic Hydrolysates of Food Proteins

Many bioactive peptides have been discovered in the enzymatic hydrolysates of various food proteins. In order to obtain active peptides, proteins used as precursors should be subjected to enzymatic hydrolysis by single or multiple enzymes under specific conditions (temperature, pH, enzyme-to-substrate ratio, hydrolysis time, etc.) for each protease. Highly active peptides can be discovered only in these optimized active enzymatic hydrolysates. It is important to optimize the hydrolysis conditions of various proteases because protein hydrolysates are present at the very beginning step of the discovery of active peptides. As reported by Nongonierma et al., an efficient way to optimize the generation of potent bioactive hydrolysates is through an approach involving multifactorial design of experiments, followed by prediction of optimal hydrolysis parameters using response surface methodology (RSM). RSM was developed by Box and collaborators in the 1950s, which can be used for experimental optimization [34]. These methods were applied in investigating DPP-IV inhibitory peptides released from milk protein and cricket protein isolates [35,36]. After hydrolysis optimization, DPP-IV inhibitory hydrolysates with the lowest half maximal inhibitory concentration (IC_50_) values were obtained, from which the DPP-IV inhibitory peptides could be further isolated and identified.

### 2.2. Fractionation and Purification of Food-Derived DPP-IV Inhibitory Peptides

**Figure 1:**
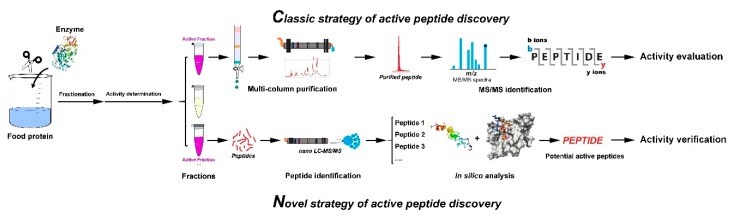
Workflow of active peptides discovery. Those “Active Fractions” in purple color were further used for active peptides purification and identification.

In general, active enzymatic hydrolysates contain peptides with a wide range of molecular weights, amino acid sequences, hydrophobicity and hydrophilicity, charge, and DPP-IV inhibitory efficacies, so that much of the classic research effort has focused on assay-directed fractionation and purification. In Figure 1, the upper part shows a classic assay-directed purification strategy. The workflow can be divided into three main steps: first, separation into crude fractions and purification of pure compounds based on different physicochemical properties (molecular weight, polarity, or charge) of each peptide; second, identification based on tandem mass (MS/MS) or Edman degradation of the purified peptides; and finally, assay of the activity of the purified peptides. Recently reported fractionation and purification methods are listed in Table 1.

#### 2.2.1. Fractionation and Purification Using Multidimensional Column Chromatography

Active enzymatic hydrolysates commonly consist of a mixture of peptides, in which the molecular size, weight distribution, hydrophobicity and other physicochemical properties of the peptides vary. Therefore, multidimensional column chromatography, including molecular exclusion chromatography, ion-exchange chromatography, reversed-phase chromatography, hydrophobic interaction chromatography, etc., should be applied to separation and purification of the peptides. Combination of two or three of the above chromatography methods should achieve peptide purification. Song et al., Huang et al., and Ji et al. combined gel filtration chromatography and C_18_ chromatography to purify DPP-IV inhibitory peptides [37,38,39]. Harnedy et al. used a C_18_ matrix solid-phase extraction (SPE) column followed by Semipreparative reversed-phase high performance liquid chromatography (SP RP-HPLC) to obtain three purified DPP-IV inhibitory peptides [40]. Sato et al. employed a gel filtration column, followed by gel filtration high-performance liquid chromatography, after which RP-HPLC was used as the final purification step, and two DPP-IV inhibitory peptides were purified and identified from natto [41]. Ion-exchange chromatography (SP Sephadex C-50 resin) combined with RP-HPLC were used to purify DPP-IV inhibitory peptides from egg yolk protein hydrolysate [31].

#### 2.2.2. Ultrafiltration for Fractionation and Purification

Membrane ultrafiltration provides an approach to fractionate mixtures of peptides according to their different molecular sizes using standard molecular weight cut-off (MWCO) membranes; this technique has been applied to the fractionation and purification of food protein-derived antihypertensive peptides [58]. Ji et al. employed ultrafiltration to separate Antarctic krill hydrolysates into different fractions with three molecular weight (MW) ranges: >10 kDa, 3–10 kDa, and <3 kDa [42]. Huang et al. reported that a porcine skin gelatin hydrolysate was first divided into three fractions with MW ranges of >2.5 kDa, 1–2.5 kDa, and <1 kDa, and both the 1–2.5 kDa and <1 kDa fractions showed great DPP-IV inhibition [59]. Zhang et al. applied three MWCO membranes, of 3, 5, and 10 kDa, to separate silver carp protein hydrolysate into fractions of four different MW ranges [43]. They used the 5 kDa MWCO membrane to obtain the < 5 kDa fraction first [44], and then the fraction exhibiting the greatest DPP-IV inhibition was further fractionated and analyzed by thin-layer chromatography (TLC) and RP-HPLC fractionation. Gallego et al. reported that two DPP-IV inhibitory peptides were purified and identified from Spanish dry-cured ham by combining ultrafiltration and size-exclusion chromatography [45]. Ultrafiltration offers the possibility of rapidly obtaining peptides of different size ranges, which is helpful for discarding inactive fractions. Normally, peptide fractions of relatively small size show better DPP-IV inhibitory activity, and these fractions are easier to fractionate further by RP-HPLC in the absence of larger peptides. In addition to the method of discarding inactive components using ultrafiltration, ethanol precipitation may be used to separate peptides of different molecular weights. Incubation in 60% ethanol (*v*/*v*) overnight allowed a hydrolysate to be separated into supernatant and pellet by centrifugation [32].

### 2.3. In Silico Approaches Applied to the Discovery of Food-Derived DPP-IV Inhibitory Peptides

As shown in the bottom section of Figure 1, a novel strategy integrating nano-liquid chromatography tandem mass spectrometry (nano-LC-MS/MS) and in silico analysis has become more efficient. This strategy consists of three steps: first, nano-LC-MS/MS can be used for peptide identification in complex mixtures like protein hydrolysates; second, molecular docking or quantitative structure activity relationship (QSAR) models based in silico analysis can be used for indicating the relationship between identified or known peptides and target proteins, which may be helpful to screen some active peptides from those identified peptides; third, potentially active peptides obtained based on virtual screening should be synthesized and determine their efficacy in vitro or in vivo.

#### 2.3.1. Use of In Silico Approaches to Predict Peptides Release

Bioinformatics is used in protein and peptide research and has become a powerful tool that can be used for in silico prediction of potentially bioactive peptides released from food proteins. For example, the BIOPEP database (http://www.uwm.edu.pl/biochemia/index.php/pl/biopep) is a bioinformatics tool enabling the detection of biologically active fragments in protein sequences [60], classification of proteins as potential sources of bioactive fragments, and simulation of protein hydrolysis to find peptides that can be released by a given enzyme or as a result of the combined action of two, three, or more enzymes [61]. An in silico study based on the BIOPEP database was applied to determine the mechanism of release of active peptides from bovine meat proteins [62].

In silico approaches based on the BIOPEP database have been used for large-scale evaluation of the potential of dietary proteins to serve as precursors of DPP-IV inhibitors [63]. In total, 34 proteins have been investigated, and more than 2000 fragments from these proteins were found to match DPP-IV inhibitory peptides reported in the literature. The occurrence frequency value (A) was calculated to evaluate the possibility of a protein serving as a bioactive peptide precursor (A = a/N, where a is the number of peptides with DPP-IV inhibitory activity in the protein sequence, and N is the number of amino acid residues in the protein chain). Thus, a higher A value suggests that the protein may more probably serve as a DPP-IV inhibitory peptide precursor. Among the 34 proteins, caseins from cow’s milk (A = 0.249), collagens from bovine meat (A = 0.380), and collagens from salmon (A = 0.305) were found to be the best potential precursors of DPP-IV inhibitors, which means that these three proteins are good DPP-IV inhibitory peptide precursors.

Using the in silico peptide prediction approach, investigators have predicted DPP-IV inhibitory peptides in food protein precursors including silver carp proteins, sodium caseinate hydrolysates, α-lactalbumin, yam dioscorin hydrolysates, β-lactoglobulin hydrolysates, and bambara bean protein hydrolysates [43,64,65,66,67,68].

In addition, an interesting study by Lacroix and Li-Chan described a method for screening α-lactalbumin-derived peptides for their interaction with DPP-IV. Decapeptide arrays spanning the entire α-lactalbumin sequence, with one amino acid frame shift between successive peptide sequences, were synthesized on cellulose membranes, and DPP-IV-binding was determined by chemiluminescence immunoassay; the DPP-IV inhibitory activity was also determined [69]. Although this method is not an in silico approach, on the basis of this cellulose membrane-assisted immobilized peptide synthesis strategy, investigators can synthesize arrays of peptides, then detect their DPP-IV binding and inhibitory ability visually and quickly.

Another in silico approach, including a peptide-alignment strategy, was developed to evaluate the DPP-IV inhibitory potential of dietary proteins. A peptide-alignment approach was used to summarize the common features of those DPP-IV inhibitory peptides having relatively low IC_50_ values (< 200 µM); the results showed that those DPP-IV inhibitory peptides have a frequent occurrence of N-terminal Tryptophan (Trp) and P1-position (the second amino acid residue of the substrate) Pro [70]. Lan et al. examined 337 synthesized dipeptides and also found that Trp frequently appeared at the N-terminus of the DPP-IV inhibitory dipeptides, and that the side chain of N-terminal Trp can interact with Phe357 of DPP-IV [71].

#### 2.3.2. Molecular Docking-Based In Silico Strategies

Molecular docking is an important tool in structural molecular biology and computer-assisted drug design. Molecular docking can be used for investigation of the interactions between ligands and receptors, and can predict the possible binding mode of a ligand with a protein; a protein with a known crystal structure can be downloaded from the RCSB Protein Data Bank (www.rcsb.org) [32,72]. The docking method can be helpful when performing virtual screening, ranking the results for a large set of compounds, and identifying the molecules with high binding scores which can “fit” or bind the receptor well from among the candidate compounds. In general, a peptide ligand pose is finally selected in terms of a docking score that represents the binding affinity. Ligands with different modes of action—including competitive, uncompetitive, and noncompetitive modes—will also show various binding score. These strategies are efficient for the discovery of active molecules. Therefore, the docking strategy has been applied recently in the screening and discovery of active food protein-derived peptides.

DPP-IV comprises some pockets where ligands can “fit”. It has been reported that there is a hydrophobic S1 pocket—Tyr631, Val656, Trp659, Tyr662, Tyr666, and Val711—and a charged S2 pocket—Arg125, Glu205, Glu206, Phe357, Ser209, and Arg358 [73]. Other literature reports state that DPP-IV has three binding pockets: S1 consists of Tyr547, Ser630, Tyr631, Val656, Trp659, Tyr662, Asn710, Val711, and His740; S2 consists of Glu205, Glu206, and Tyr662; and S3 consists of Ser 209, Arg358 and Phe357 [74]. As Metzler et al. reported, Ser630, His740, and Asn708 are the catalytic triad [75]; David et al. also reported that catalytic triad consists of Ser624, Asn702, and His734 in mouse DPP-IV [76]. In any case, the binding sites of DPP-IV described in these two reports are similar. In Table 2, the potential binding sites of DPP-IV inhibitory peptides are listed according to the findings of molecular docking analysis. Those studies use different DPP-IV (with different protein data bank (PDB) code) for molecular docking. What the reason is that different proteins might be selected from PDB based on the compound the DPP-IV complexed. To some extent, the complex compound is similar with the peptides which need to be screened by molecular docking. Mudgil et al. reported the identification of 471 and 317 peptides from two hydrolysates using nano-LC-MS/MS, followed by the application of the Peptide Ranker web server and Pepsite2 software to screen and examine the DPP-IV inhibitory activity and the possible binding site in the DPP-IV in silico. Twenty peptides among the 888 peptides identified with a Peptide Ranker score > 0.8 were considered to be potential bioactive peptides, and, after Pepsite2 screening, 18 peptides were considered to be DPP-IV inhibitory peptides. The Pepsite2 program gives the Reactive residue in a peptide together with the Bound residues of DPP-IV [77]. For example, peptide Ala-Glu-Trp-Leu-His-Asp-Trp-Lys-Leu (AEWLHDWKL)showed a Peptide Ranker score of 0.84, and Pepsite2 showed that it could bind Tyr48, Tyr547, Trp627, Trp629, Tyr631, Tyr666, and Tyr752 through its W_3_L_4_H_5_K_8_L_9_ amino acid residues. However, although screening and activity examination based on docking software are efficient, in vitro or in vivo determination of activity should be carried out after virtual screening.

Liu et al. utilized a protein database searching method based on tandem mass (MS/MS) spectra to identify 50 peptides in the DPP-IV inhibitory fraction derived from *Ruditapes philippinarum* (Manila clam) flesh hydrolysate [32]. In order to find active peptides rapidly in this 50-peptide set, Discovery Studio software was employed for the molecular docking analysis. Among the 50 peptides, four peptides were found to “fit” into the pockets through hydrogen bonding, charge, polar, or van der Waals interactions. Peptides that occupy all the pockets of DPP-IV well should have a lower CDOCKER energy value (CDOCKER is a molecular dynamics simulated-annealing-based algorithm), which means that the peptide may show high DPP-IV inhibitory activity. However, owing to their binding modes, some peptides showed the highest DPP-IV inhibitory activity despite the fact that their CDOCKER energy values were not the lowest. For instance, the CDOCKER energy values of Phe-Ala-Gly-Asp-Asp-Ala-Pro-Arg (FAGDDAPR), Leu-Ala-Pro-Ser-Thr-Met (LAPSTM), Phe-Ala-Gly-Asp-Asp-Ala-Pro-Arg-Ala (FAGDDAPRA), and Phe-Leu-Met-Glu-Ser-His (FLMESH) were −88.5, −85.6, −80.2, and −75.5 kcal/mol, respectively. FAGDDAPR showed the lowest CDOCKER energy, while LAPSTM showed the highest inhibitory activity (IC_50_ = 140.8 µM). FAGDDAPR could bind Arg125, Arg358, and Phe357 in the S2 pocket very well, while LAPSTM could form five hydrogen bonds with Ser630, Arg125, Ser209, and Try662, and form a π–π interaction with Tyr666. It seemed that LAPSTM occupied the pockets better than FAGDDAPR. Therefore, using only computer-assisted docking to screen active peptides is insufficient: activity verification is necessary for all virtually screened peptides [32]. Using a similar molecular docking strategy, Wang et al. found a DPP-IV inhibitory peptide (Leu-Gln-Ala-Phe-Glu-Pro-Leu-Arg, LQAFEPLR) from oats, and the subsequent DPP-IV inhibition assay confirmed its activity, with an IC_50_ value of 103.5 µM [81].

#### 2.3.3. Quantitative Structure Activity Relationship (QSAR) Model-Based In Silico Analysis

QSAR is a method for building computational or mathematical models which attempts to find a correlation between structure and function using a chemometric technique [82]. In terms of the discovery of active peptides, structure refers to the amino acid sequences of the peptides, and function refers to the binding affinity, activity, etc. Various QSAR methods have been established and serve as a powerful predictive tool for active peptide discovery. Nongonierma et al. used competitive DPP-IV inhibitory peptides with IC_50_ values ranging from 3.5 to 3216.8 µM to build a QSAR model [83]. The QSAR model could be utilized to predict the DPP-IV inhibitory potency of milk protein-derived peptides. Two kinds of scale, a z-scale, including the parameters hydrophilicity, size, and charge, and a v-scale, including the parameters van der Waals volume, net charge index, and hydrophobic parameter of side chains, were used for the amino acid descriptors, following which peptide descriptors could be generated. After that, two QSAR models linking the peptide descriptors and DPP-IV inhibitory IC_50_ values were established. As a result, although neither QSAR model accurately predicted the DPP-IV inhibitory IC_50_ values of the peptides, the ranking of the peptides was consistent with the verified experimental values [83]. QSAR-based in silico analysis also demonstrated that a hydrophobic N-terminal amino acid was related to DPP-IV inhibitory activity; additionally, an Ile residue in the P2-postion (the first amino acid residue of the substrate) and a Pro residue in the P1 position were frequently observed. A method combining QSAR and molecular docking was applied to find novel DPP-IV inhibitory peptides from the analogs of Ile-Pro-Ile, investigating the binding interactions between DPP-IV and peptides, the mode of inhibition and the stability of the active peptides [79].

We consider that compared with experimental methods, the advantages of in silico methods are efficient and convenient when predicting possible peptide composition from precursors in the presence of specific protease activity [84]; the potential biological function of sequenced peptides could also be evaluated in silico by searching bioinformatics databases or using molecular docking methods [85]. However, limitations also exist: only those protein database existing receptors structures can be used for in silico analysis; in nano-LC-MS/MS based identification of protein hydrolysates, some peptides might not be identified or wrongly assigned; and molecular docking does not reflect the actual physical process of binding, and, in some cases, prevents the correct identification of potential active candidates due to the receptor conformational space. In addition, the in vitro or in vivo bioactivity of these putative DPP-IV inhibitory peptides needs to be verified; can we obtain these theoretical food-derived peptides only by consuming food daily? What is known about their bioavailability and in vivo activity after gastrointestinal digestion?

## 3. DPP-IV Inhibitory Peptides Derived from Food Protein Hydrolysates

Ile-Pro-Ile (IPI) has been reported as the most potent DPP-IV inhibitory peptide (IC_50_ = 5 μM), and it is also present in the primary sequence of several food proteins [70]. For instance, Ile-Pro-Ile is present from Ile26 to Ile28 in bovine κ-casein. We have listed the DPP-IV inhibitory peptides from food protein hydrolysates discovered in the past three years in Table 3.

As a substrate of DPP-IV, Pro at the P1-position is the preferred amino acid residue, and Alanine (Ala), Glycine (Gly), hydroxyproline (Hyp), and other small residues are also accepted by the hydrophobic pocket of DPP-IV [63]. At the P2-position, various hydrophobic, basic, or neutral amino acid residues, or amino acid residues with bulky side chains, such as Trp or Tyrosine (Tyr), may enhance the binding ability. Nongonierma et al. reported that among the 19 dipeptides with N-terminal Trp, only Trp-Asp was not a DPP-IV inhibitor [87]. Peptides with Pro at the P1-position and/or N-terminal Trp might exhibit high DPP-IV inhibitory activity [70]. In addition, the P1′-position (the third amino acid residue of the substrate) must not be Proline (Pro) or Hyp. Therefore, for these DPP-IV inhibitory peptides, the amino acid residues at the P1, P2, and P1′-positions play important roles in affecting the peptides’ DPP-IV inhibitory activity.

## 4. Distribution of Known DPP-IV Inhibitory Peptides

**Figure 2:**
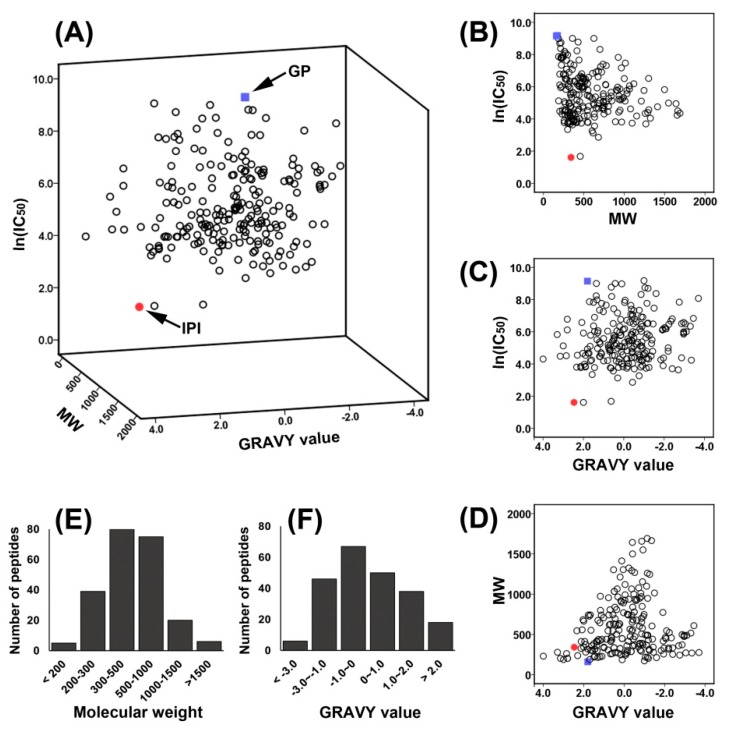
Distribution of known DPP-IV inhibitory peptides. Red dot: tripeptide IPI. Blue square: dipeptide Gly-Pro (GP). (**A**) 3D distribution of 222 peptides based on their IC_50_, MW, and GRAVY values; (**B**) distribution of 222 peptides according to IC_50_ and MW; (**C**) distribution of peptides according to IC_50_ and GRAVY values; (**D**) distribution of peptides according to MW and GRAVY values; and (**E**) and (**F**) MW and GRAVY value distributions of the 222 peptides, respectively, based on the total number of peptides.

The molecular weight, hydrophobicity, and other physicochemical properties of bioactive peptides may influence their bioavailability, target binding mode, or bioactivity. In total, 222 food-derived DPP-IV inhibitory peptides discovered in recent decades are listed (Table S1), with IC_50_ values ranging from 5 to 9690 µM. We have produced a 3D scatter plot based on these peptides’ molecular weights (MWs), IC_50_ values, and grand average of hydropathicity (GRAVY) values to visualize the distribution and classification of these peptides. As shown in Table S1 and Figure 2, among the reported DPP-IV inhibitory peptides, over 88.4% have a MW lower than 1000 Da, and more than 50% have MWs lower than 500 Da (Figure 2E). The GRAVY value was employed to evaluate the hydrophilic and hydrophobic character of these peptides: approximately 53% of these peptides are hydrophilic with a GRAVY value lower than 0, with 47% of these peptides being hydrophobic (Figure 2F). About 70% of the peptides have IC_50_ values from 50 to 1000 µM, which are clustered as shown in Figure 2A. In Figure 2B, among the peptides with MWs lower than 500 Da, nearly 80% of peptides have IC_50_ lower than 1000 µM. In Figure 2C,D, it appears that the GRAVY values of the peptides are distributed uniformly. Based on the classification and distribution, we considered that the peptides’ MWs and GRAVY may be related to their DPP-IV inhibitory activity.

## 5. In Vivo Activity and Bioavailability of DPP-IV Inhibitory Peptides

In most of the recent investigations, the DPP-IV inhibitory activities of peptides are usually evaluated by in silico or in vitro assays. However, in vivo experiments involving activity assays are necessary to demonstrate the physiological effect of the peptides. There are studies focusing on the in vivo DPP-IV inhibitory activity [5]. Hsieh et al. studied the in vivo DPP-IV inhibitory activity of Atlantic salmon skin gelatin hydrolysate using rats with streptozotocin (STZ)-induced diabetes [88]. After five weeks of oral administration of FSGH, the blood glucose levels of the diabetic rats decreased during an oral glucose tolerance test, and plasma DPP-IV activity was inhibited, while plasma GLP-1 and insulin levels were increased. In addition, Wang’s investigation showed that Tilapia skin gelatin hydrolysate (TSGH) has high in vitro DPP-IV inhibitory activity; after 30 days’ daily administration of TSGH, glucose tolerance in rats with STZ-induced diabetes improved, and GLP-1 and insulin secretion were enhanced [53]. Although food-derived protein hydrolysates display DPP-IV inhibitory and antidiabetic efficacy both in vitro and in vivo, the oral bioavailability of hydrolytic peptides is commonly very low owing to extensive hydrolysis of the peptides in the gastrointestinal tract by peptidases in the stomach, small intestinal, and brush border, as well as low cellular uptake of these peptides.

Foltz et al. considered that it is meaningful to propose in vivo efficacy for bioactive peptides when the peptide exhibits reasonable proteolytic stability and physiologically relevant absorption, distribution, metabolism, and excretion (ADME) profiles [89]. Therefore, if the DPP-IV inhibitory activity of peptides demonstrated in vitro is to translate into the in vivo context, the bioavailability of the peptides must be considered [13]. A report concerning the bioavailability of angiotensin-converting enzyme-inhibitory tripeptides showed that Ile-Pro-Pro could be found intact in the circulation after individuals consumed a beverage enriched with the lactotripeptide Ile-Pro-Pro [90]. Nevertheless, the bioavailability of DPP-IV inhibitory peptides has not been well studied to date, and it is still not very clear whether these peptides can reach the DPP-IV target sites intact and exert their physiological effect. Dipeptides and tripeptides are considered to be able to cross the intestinal endothelium and reach the systemic circulation intact [91]. Lacroix et al. investigated the stability and cellular transport of milk-derived peptides with in vitro DPP-IV inhibitory activity [92]. However, in the Caco-2 cell monolayer model system, only a small percentage—ranging from 0.05 to 0.47%—of the DPP-IV inhibitory peptides, Leu-Lys-Pro-Thr-Pro-Glu-Gly-Asp-Leu (LKPTPEGDL), Leu-Pro-Tyr-Pro-Tyr (LPYPY), Ile-Pro-Ile-Gln-Tyr (IPIQY), Ile-Pro-Ile (IPI), and Trp-Arg (WR), could cross the monolayer intact. Food-derived DPP-IV inhibitory peptides may be susceptible to degradation by the intestinal brush border membrane enzymes, and such factors may alter their DPP-IV inhibitory activity in vivo. However, although their ability to cross the membranes is limited, some of the DPP-IV inhibitory peptides display high in vivo antidiabetic activities. Meanwhile, there is another viewpoint that DPP-IV inhibitory peptides derived from food proteins may serve as endogenous inhibitors of DPP-IV in the proximal small intestine, but not in the plasma [93].

Therefore, in future work, evaluation of the in vivo DPP-IV inhibitory activity of peptides should be carried out after screening for in vitro DPP-IV inhibitory activity. Moreover, stability in the gastrointestinal tract, ADME profiles, and bioavailability should be investigated as well, to help us understand how these food-derived DPP-IV inhibitory peptides exert their glycemia regulatory effect after oral administration.

## 6. Conclusions

Safe and convenient methods to prevent diseases, especially chronic and metabolic disorders such as T2DM, hypertension, etc., are widely sought. Diets rich in specific bioactive ingredients, including food protein-derived peptides, have potential application in the prevention and management of T2DM. Food-derived peptides may be a complementary strategy to help regulate glycemia in diabetic or prediabetic individuals. Thus, it is necessary to find clinical evidence of the effect of food-derived peptides in regulating blood glucose. It is also necessary to develop powerful strategies by which to discover more food-derived DPP-IV inhibitory ingredients, not only purified active peptides, but hydrolysates or peptide mixtures, which may show greater safety and potency.

## Figures and Tables

**Table 1:** Recently reported examples of methods on DPP-IV inhibitory peptides fractionation and purification.

Protein Source	Fractionation Method	Resin/Material	Condition	Ref.
Manila clam flesh, papain hydrolysate	1. Ethanol precipitation 2. RP-HPLC *	1. – * 2. Waters Xbridge C_18_	1. 60% final concentration 2. Methanol linear gradient elution	[32]
Mare whey protein, papain hydrolysate	1. Ultrafiltration 2. Gel permeation chromatography 3. RP-HPLC	1. 10 kDa MWCO * 2. GE Sephadex G-25 3. Agilent XDB-C_18_	1. – 2. Deionized water isocratic elution 3. Acetonitrile linear gradient elution	[37]
Tuna cooking juice, protease XXIII hydrolysate	1. Gel filtration chromatography 2. RP-HPLC	1. Sephadex G-25 2. Zorbax Eclipse Plus C_18_	1. H_2_O isocratic elution 2. Acetonitrile linear gradient elution	[38]
Antarctic krill, animal proteolytic enzyme hydrolysate	1. Ultrafiltration 2. Gel permeation chromatography 3. RP-HPLC	1. MWCO membrane (5, 3, 0.1 kDa) 2. GE Sephacryl-100 3. Waters YMC-Pack ODS-AQ C_18_	1. – 2. 0.15 M NaCl isocratic elution 3. 5% Methanol isocratic elution	[39]
*Palmaria palmata* protein, Corolase PP * hydrolysate	1. SPE column * 2. semi-preparative RP-HPLC	1. Strata-X C_18_ 2. C_18_	1. Acetonitrile stage elutions 2. Acetonitrile linear gradient elution	[40]
Fermented soybean, water-soluble extract	1. Ultrafiltration 2. Gel permeation chromatography 3. Gel filtration HPLC 4. RP-HPLC	1. 3 kDa MWCO 2. Tosoh HW-40S 3. Superdex peptide 10/300 GL 4. Tosoh ODS-80Ts	1. – 2. H_2_O isocratic elution 3. Tris-HCl buffer 4. Acetonitrile linear gradient elution	[41]
Antarctic krill, Corolase PP, alcalase, flavourzyme, and papain	1. Ultrafiltration 2. Gel permeation chromatography 3. Ion-exchange chromatography 4. RP-HPLC	1. MWCO membrane (10, 3 kDa) 2. GE Sephacryl S-100 3. GE Q-Sepharose Fast Flow 4. Waters YMC-Pack ODS-AQ C_18_	1. – 2. 0.15 M NaCl isocratic elution 3. NaCl linear gradient elution 4. Acetonitrile linear gradient elution	[42]
Silver Carp, neutrase-generated hydrolysate	1. Ultrafiltration 2. Thin-layer chromatography 3. RP-HPLC	1. MWCO membrane (10, 5, and 3 kDa) 2. Silica gel TLC plate * 3. C_18_	1. – 2. Chloroform/methanol/25% ammonia 3. Acetonitrile linear gradient elution	[43]
Goat milk casein, trypsin hydrolysate	1. Ultrafiltration 2. Semi preparation TLC 3. RP-HPLC	1. MWCO membrane 5 kDa 2. Silica gel TLC plate 3. C_18_	1. – 2. Chloroform/methanol/25% ammonia 3. Acetonitrile linear gradient elution	[44]
Spanish dry-cured ham,	1. Ethanol precipitate 2. Ultrafiltration 3. Size-exclusion chromatography	1. – 2. MWCO membrane 3, 1 kDa 3. Sephadex G25	1. 3 volumes of ethanol 2. – 3. 0.01 N HCl isocratic elution	[45]
Barbel Alcalase hydrolysate	1. Gel permeation chromatography 2. RP-HPLC 3. RP-HPLC	1. GE superdex peptide 10/300 2. TRACER-Excel 120 3. HALO Peptide ES-C_18_	1. Ammonium acetate buffer isocratic elution 2. Acetonitrile linear gradient elution 3. Acetonitrile linear gradient elution	[46]
Salmon gelatin, Alcalase 2.4 L hydrolysate	Semipreparative RP-HPLC	C_18_	Acetonitrile linear gradient elution	[47]
Wheat gluten, Ginger protease hydrolysate	Size-exclusion chromatography	Superdex Peptide HR 10/30	20% Acetonitrile isocratic elution	[29]
Gouda-type cheese water-soluble extracts	RP-HPLC	Protein/peptide C_18_	Acetonitrile linear gradient elution	[48]
Trypsin-treatedβ-Lactoglobulin	RP-HPLC	Ethylene Bridged Hybrid (BEH) 130 PREP C_18_	Acetonitrile linear gradient elution	[49]
Whey protein, trypsin hydrolysate	SP RP-HPLC	Prep Nova-Pack HR C_18_ column	Acetonitrile linear gradient elution	[50]
*Palmaria palmata* alcalase, flavourzyme and corolase PP hydrolysate	Gel permeation chromatography	TSK G2000 SW	Acetonitrile isocratic elution	[51]
Salmon skin gelatin, alcalase, bromelain, Flavourzyme hydrolysate	1. Ultrafiltration 2. RP-HPLC	1. MWCO membrane 2.5 and 1 kDa 2. Zorbax Eclipse Plus C_18_	1. – 2. Acetonitrile linear gradient elution	[52]
Fish skin gelatin, Flavourzyme hydrolysate	Ultrafiltration	MWCO membrane 2.5, 1.5 kDa	–	[53]
Collagen (from pig, cattle, fish, and chicken), collagenase hydrolysate	Gel permeation chromatography	Superdex peptide column	Tris-HCl buffer contain 150 mM NaCl isocratic elution	[54]
Whey protein, thermoase hydrolysate	1. Size-exclusion chromatography 2. RP-HPLC	1. Superdex peptide 10/300 GL 2. Jupiter C_12_	1. Tris-HCl buffer isocratic elution 2. Acetonitrile linear gradient elution	[55]
Rice bran, Umamizyme G hydrolysate	1. Gel filtration chromatography 2. preparative HPLC	1. HiLoad 26/60 Superdex 30 prep grade column 2. Inertsil ODS-3 column	1. Tris-HCl buffer contain 150 mM NaCl isocratic elution 2. Acetonitrile linear gradient elution	[56]
Bovine whey protein, pepsin hydrolysate	1. Cation-exchange chromatography 2. Size-exclusion chromatography 3. RP-HPLC	1. Mono S 5/50 GL cation-exchange column 2. Superdex peptide 10/300 GL 3. Jupiter C_12_	1. Sodium acetate buffer, with linear gradient and stage elution 2. Tris-HCl buffer isocratic elution 3. Acetonitrile linear gradient elution	[57]
Atlantic salmon, Alcalase, Flavourzyme, Corolase PP, Promod hydrolysate	SP RP-HPLC	C_18_	Acetonitrile linear gradient elution	[28]

*: –, there is no resin or material involved. RP-HPLC, Reversed phase high-performance liquid chromatography. MWCO, Molecular weight cut off. Corolase PP, food-grade porcine pancreatic proteolytic preparation. SPE column, Solid Phase extraction column. TLC, Thin layer chromatography.

**Table 2:** Recently reported potential binding sites of DPP-IV inhibitory peptides based on molecular docking analysis.

No.	Sequences	Potential Binding Sites	Protein Data Bank (PDB) Code	Software	Ref.
1	Trp-Ser-Gly	Lys122, Trp124, Arg125, Trp201, Glu205, Tyr547, Trp629, Ser630, Tyr631, Tyr662, Tyr666, Asp709, Asn710, Asp739, His740, Gly741	2AJB	Molegro Virtual Docker v.6.0.0 software	[46]
2	Phe-Ser-Asp	Arg125, Glu205, Glu206, Ser209, Phe357, Arg358, Tyr547, Trp629, Ser630, Tyr631, Tyr662, Tyr666, His740			
3	Ile-Ala-Val-Pro-Thr-Gly-Val-Ala	Glu205, Glu206, Ser209, Arg358,	4PNZ	VEGA	[78]
4	Leu-Thr-Phe-Pro-Gly-Ser-Ala-Glu-Asp	Glu205, Glu206, Arg125, Arg356, Arg358, Arg429, Tyr547, Trp629, His740			
5	Ala-Pro	Thr94, Phe95, Asp104, Try105	2QT9	Discovery Studio 4.5 Auto Dock 4.2.6	[39]
6	Ile-Pro-Ala	Glu91, Asn92, Ser93, Thr94, Phe95, Asp96, Ser101, Ile102			
7	Phe-Ala-Gly-Asp-Asp-Ala-Pro-Arg	Arg125, Phe357, Arg358, Lys554	1WCY	Discovery Studio 4.0	[32]
8	Leu-Ala-Pro-Ser-Thr-Met	Arg125, Glu205, Ser209, Tyr662, Ser630, Tyr666			
9	Phe-Ala-Gly-Asp-Asp-Ala-Pro-Arg-Ala	Ser209, Tyr547, Tyr585			
10	Phe-Leu-Met-Glu-Ser-His	Arg125, Arg358			
11	Ala-Glu-Trp-Leu-His-Asp-Trp-Lys-Leu	Tyr48, Tyr547, Trp627, Trp629, Tyr631, Tyr666, Tyr752	4A5S	Pepsite2 software	[77]
12	Ala-Val-Val-Ser-Pro-Leu-Lys-Pro-Cys-Cys	Tyr547, Val653, Trp627, Trp629, Tyr631, Tyr666, Ile752, Tyr752, Met755			
13	Cys-Phe-Leu-Pro-Leu-Pro-Leu-Leu-Lys	Phe357, Tyr547, Tyr585, Trp629, Tyr631, Tyr666, Tyr670, Tyr752			
14	Asp-Asn-Leu-Met-Pro-Gln-Phe-Met	Glu206, Ser209, Phe357, Pro550, Tyr547, Trp629, Ser630, Tyr631, Tyr666, Tyr670			
15	Phe-Cys-Leu-Pro-Leu-Pro-Leu-Leu-Lys	Tyr48, Phe357, Tyr547, Trp627, Trp629, Tyr631, Tyr666, Tyr670, Tyr752			
16	Phe-Met-Phe-Phe-Gly-Pro-Gln	Phe357, Tyr547, Pro550, Trp627, Trp629, Tyr666, Tyr670, Tyr752			
17	Gly-Met-Ala-Gly-Gly-Pro-Pro-Leu-Leu	Phe357, Tyr547, Pro550, Trp629, Tyr666, Tyr670, His740, Gly741, Tyr752			
18	His-Cys-Pro-Val-Pro-Asp-Pro-Val-Arg-Gly-Leu	Tyr48, Phe357, Tyr547, Cys551, Trp627, Ser630, Tyr631, Val653, Tyr666, Gly741, His748, Tyr752			
19	Lys-Phe-Gln-Trp-Gly-Tyr	Tyr547, Trp627, Ser630, Val653, Tyr666, Tyr752			
20	Leu-Leu-Pro-Ala-Pro-Pro-Leu-Leu	Phe357, Val546, Tyr547, Trp627, Trp629, Ser630, Tyr666, Tyr752			
21	Leu-Thr-Met-Pro-Gln-Trp-Trp	Tyr48, Trp627, Trp629, Ser630, Val653, Ile703, His740, Ile742, Tyr752, Met755			
22	Met-Met-His-Asp-Phe-Leu-Thr-Leu-Cys-Met	Tyr48, Phe357, Val546, Tyr547, Cys551, Tyr585, Trp627, Ser630, Tyr631, Tyr666, Tyr752			
23	Met-Ser-Lys-Phe-Leu-Pro-Leu-Pro-Leu-Met-Phe-Tyr	Tyr48, Phe357, Tyr547, Tyr585, Trp627, Trp629, Tyr666, Tyr670, Gly741, Tyr752			
24	Ser-Gln-Asp-Trp-Ser-Phe-Tyr	Ser209, Phe357, Tyr547, Pro550, Tyr585, Tyr631, Tyr666, Tyr670			
25	Trp-Gly-Leu-Trp-Asp-Asp-Met-Gln-Gly-Leu	Tyr48, Tyr547, Trp627, Trp629, Tyr631, Tyr666, His740, His748, Tyr752			
26	Trp-Asn-Trp-Gly-Trp-Leu-Leu-Trp-Gln-Leu	Tyr48, Glu205, Glu206, Phe357, Tyr547, Trp627, Trp629, Tyr631, Val653, Tyr666, Ile703, Ile742, His748, Ile751, Tyr752, Met755			
27	Tyr-Trp-Tyr-Pro-Pro-Lys	Tyr48, Trp627, Trp629, Gly741, His748, Tyr752			
28	Tyr-Trp-Tyr-Pro-Pro-Gln	Phe357, Tyr547, Pro550, Tyr631, Tyr666, Tyr670			
29	Thr-Leu-Met-Pro-Gln-Trp-Trp	Tep48, Val546, Trp627, Gly628, Trp629, Ser630, His748, Tyr752			
30	Met-Pro-Ser-Lys-Pro-Pro-Leu-Leu	Tyr48, Phe357, Tyr547, Trp627, Trp629, Tyr631, Tyr666, His748, Tyr752			
31	Ala-Val-Val-Ser-Pro-Leu-Lys-Pro-Cys-Cys	Tyr547, Trp627, Trp629, Tyr631, Val653, Tyr666, Ile703, Ile742, Ile751, Tyr752, Met755			
32	Ala-Pro-Ala	Arg125, Glu205, Glu206, Tyr662	1ORW	Sybyl software 8.1	[79]
33	Ala-Pro-Phe	Arg125			
34	Ala-Pro-Arg	Arg125, Glu205, Glu206, Ser630, Tyr662			
35	Ile-Pro-Ala	Arg125, Glu205, Glu206, Tyr662			
36	Lys-Pro-Ala	Arg125, Glu205, Glu206, Tyr662			
37	Phe-Pro-Phe	Arg125, Glu205, Glu206			
38	Phe-Pro-Ile	Arg125, Glu205, Glu206, Tyr662			
39	Phe-Pro-Trp	Arg125, Glu205, Glu206, Tyr662			
40	Ile-Pro-Phe	Arg125, Glu205, Glu206, Ser630, Tyr662			
41	Ile-Pro-Trp	Arg125, Glu205, Glu206, Tyr662			
42	Trp-Pro-Phe	Arg125, Glu205, Glu206, Tyr662			
43	Trp-Pro-Ile	Arg125, Glu205, Glu206, Tyr662			
44	Trp-Pro-Trp	Arg125, Glu205, Glu206, Tyr662			
45	Tyr-Pro-D-Ala-NH_2_ *	Glu205, Glu206, Tyr547, Ser630, His740, Asp708	5I7U	MVD v. 6.0.1.	[80]
46	Ala-Lys-Ser-Pro-Leu-Phe	Glu191, Asp192, Leu235, Arg253	3W2T	Docking Server	[30]
47	Gln-Thr-Pro-Phe	Asp192, Thr251, Arg253, Val252, Val254			
48	Phe-Glu-Glu-Leu-Asn	Glu191, Asp192, Pro249			
49	Leu-Ser-Lys-Ser-Val-Leu	Asp192, Glu237, Lys250, Thr251, Arg253			
50	Leu-Gln-Ala-Phe-Glu-Pro-Leu-Arg	Phe357, Arg429, Tyr456, Asp556, Tyr585, Trp629, Ser630, Tyr662, His740	1X70	Auto Dock Vina	[81]
51	Glu-Phe-Leu-Leu-Ala-Gly-Asn-Asn-Lys	Arg125, Ser209, Arg358, Arg429, Tyr456, Tyr547, Tyr585, Trp629, Ser630, His740			

* Not derived from food.

**Table 3:** DPP-IV inhibitory peptides discovered between 2016 and 2018.

Protein Source	Sequence	IC_50_ (μM)	Ref.
Ile-Pro-Ile (IPI) analogs	Ala-Pro-Ala	43.3	[79]
	Ala-Pro-Phe	65.8	
	Ala-Pro-Arg	119.7	
	Ile-Pro-Ala	28.3	
	Lys-Pro-Ala	74.5	
	Phe-Pro-Phe	247	
	Phe-Pro-Ile	45.2	
	Phe-Pro-Trp	54.9	
	Ile-Pro-Phe	47.3	
	Ile-Pro-Trp	175.3	
	Trp-Pro-Phe	159.8	
	Trp-Pro-Thr	133	
	Trp-Pro-Trp	120.1	
Soybean glycinin	Ile-Ala-Val-Pro-Thr-Gly-Ala	106	[78]
Lupin seed β-Conglutin	Leu-Thr-Phe-Pro-Gly-Ser-Ala-Glu-Asp	228	
Yam dioscorin	Arg-Arg-Asp-Tyr	930	[66]
	Ile-His-Phe	3770	
	Lys-Arg-Ile-His-Phe	4110	
	Arg-Leu	1200	
	Gly-Pro-Ala	2870	
	Met-Gly-Ser-Phe	2120	
	Asp-Pro-Phe	1540	
β-Casein	Leu-Pro-Val-Pro-Gln	43.8	[83]
	Val-Pro-Gly-Glu-Ile-Val-Glu	224.5	
	Tyr-Pro-Phe-Pro-Gly-Pro	749.2	
	Leu-Pro-Gln-Asn-Ile-Pro-Pro-Leu-Thr	205.2	
	Ile-Pro-Pro-Leu-Thr-Gln-Thr	465.1	
	Thr-Pro-Val-Val-Val-Pro-Pro	1408.9	
	Tyr-Pro-Val-Glu-Pro-Phe	124.7	
	Leu-Pro-Leu-Pro-Leu-Leu	371.5	
	Gln-Pro-His-Gln-Pro-Leu-Pro-Pro-Thr	1754.8	
	Gln-Pro-Leu-Pro-Pro-Thr	1013.8	
	Ile-Pro-Pro-Leu	428.9	
	Leu-Pro-Pro	563.3	
Milk protein	Val-Pro	380.3	
	Arg-Pro	657.2	
	Phe-Pro	682.5	
	His-Pro	902.8	
Lactoferrin	Ile-Pro-Met	69.5	
	Ile-Pro-Ser-Lys	406.8	
Barbel	Trp-Ser-Gly	209.9	[46]
	Phe-Ser-Asp	275.1	
Silver Carp	Leu-Pro-Ile-Ile-Asp-Ile	105.4	[43]
	Ala-Pro-Gly-Pro-Ala-Gly-Pro	229.1	
Antarctic krill	Lys-Val-Glu-Pro-Leu-Pro	1071.9*	[42]
	Pro-Ala-Leu	2943.1*	
Manila clam	Phe-Ala-Gly-Asp-Asp-Ala-Pro-Arg	168.7	[32]
	Leu-Ala-Pro-Ser-Thr-Met	140.8	
	Phe-Ala-Gly-Asp-Asp-Ala-Pro-Arg-Ala	393.3	
	Phe-Leu-Met-Glu-Ser-His	> 500	
Salmon gelatin	Gly-Gly-Pro-Ala-Gly-Pro-Ala-Val	8139.1	[47]
	Gly-Pro-Val-Ala	264.7	
	Pro-Pro	4343.5	
	Gly-Phe	1547.1	
β-Lactoglobulin	Asn-Leu-Gly-Ile-Ile-Leu-Arg	86.3	[37]
	Thr-Gln-Met-Val-Asp-Glu-Glu-Ile-Met-Glu-Lys-Phe-Arg	68.8	
Wheat gluten	Gln-Pro-Gln	79.8	[29]
	Gln-Pro-Gly	70.9	
	Gln-Pro-Phe	71.7	
	Leu-Pro-Gln	56.7	
	Ser-Pro-Gln	78.9	
Camel milk	Ile-Leu-Asp-Lys-Glu-Gly-Ile-Asp-Tyr	347.8	[27]
	Ile-Leu-Asp-Lys-Val-Gly-Ile-Gln-Tyr	321.5	
	Ile-Leu-Glu-Leu-Ala	721.1	
	Leu-Leu-Gln-Leu-Glu-Ala-Ile-Arg	177.8	
	Leu-Pro-Val-Pro	87.0	
	Met-Pro-Val-Gln-Ala	93.3	
	Ser-Pro-Val-Val-Pro-Phe	214.1	
	Tyr-Pro-Val-Glu-Pro-Phe	138.0	
Fermented soybean	Lys-Leu	159.8*	[41]
	Leu-Arg	2083.6*	
Synthetic	Tyr-Pro-Leu	364.6	[86]
	Tyr-Pro-Gly	174.0	
Atlantic salmon	Gly-Pro-Ala-Val	245.6	[28]
	Phe-Phe	546.8	
	Val-Cys	5413.4	
Globulin	Leu-Gln-Ala-Phe-Glu-Pro-Leu-Arg	103.5	[81]

* IC_50_ (μM) value was converted from mg/mL.

## Supplementary Materials

Supplementary materials can be found at http://www.mdpi.com/1422-0067/20/3/463/s1.

## Abbreviations

T2DM	Type 2 diabetes mellitus
DPP-IV	Dipeptidyl peptidase IV
GLP-1	Glucagon-like peptide 1
GIP	Glucose-dependent insulinotropic polypeptide
MW	Molecular weight
MWCO	Molecular weight cutoff
QSAR	Quantitative structure activity relationship
GRAVY	Grand average of hydropathicity
His	Histidine
Asp	Aspartic acid
Arg	Arginine
Phe	Phenylalanine
Ala	Alanine
Cys	Cysteine
Gly	Glycine
Gln	Glutamine
Glu	Glutamic acid
Lys	Lysine
Leu	Leucine
Met	Methionine
Asn	Asparagine
Ser	Serine
Tyr	Tyrosine
Thr	Threonine
Ile	Lsoleucine
Trp	Tryptophan
Pro	Proline
Val	Valine
