# Supplementary material for: Discovery of Food-Derived Dipeptidyl Peptidase IV Inhibitory Peptides: A Review
Source: Int J Mol Sci. 2019 Jan 22;20(3):463. doi: 10.3390/ijms20030463 (PMC6387223; doi:10.3390/ijms20030463)
Supplement: Supplementary file 1 [file ijms-20-00463-s001.pdf]

International Journal of
Molecular Sciences

MDPI

Table 1. IC_{50}, molecular weight and GRAVY value of DPP-IV inhibitory peptides.

Sequence	IC_{50} (μM)	References	Molecular Weight	GRAVY Value
IPI	5	[1]	341.4509	2.467
LPL	5	[1]	341.4509	2.000
KVLP	5.3 *	[1]	455.5981	0.625
LPVPQK	17.4 *	[1]	680.8533	−0.433
VPLGTQ	22.8 *	[1]	613.7119	0.300
VPYPQ	26.1 *	[1]	602.688	−0.760
IPA	28.3	[2]	299.3702	1.567
IPIQY	35.2	[2]	632.7576	0.520
WR	37.8	[2]	360.4161	−2.700
INNQFLPYPY	40.1	[2]	1268.449	−0.520
WK	40.6	[2]	332.4027	−2.400
GPGA	41.9	[2]	300.3163	−0.150
PLLQ	42.0 *	[1]	469.5816	0.625
LKPTPEGDLE	42	[2]	1098.227	−1.110
APA	43.3	[3]	257.2895	0.667
ILAP	43.4	[2]	412.5343	2.125
WL	43.6	[2]	317.388	1.450
LPVPQ	43.8 *	[1]	552.6783	0.260
WP	44	[2]	301.3452	−1.250
IPAVF	44.7	[2]	545.6853	2.340
LKPTPEGDL	45	[2]	969.1113	−0.844
FPI	45.2	[3]	375.4679	1.900
LPQNIPPL	46	[2]	891.0893	0.038
GPFP	46.4 *	[1]	416.4813	−0.200
VPL	47	[2]	327.424	2.133
IPF	47.3	[3]	375.468	1.900
TH	49	[2]	256.2614	−1.950
GPAE	49.6	[2]	372.3782	−0.925
LLAP	53.7	[2]	412.5343	1.950
FPW	54.9	[3]	448.5218	0.100
LPQ	56.7	[2]	356.4222	−0.433
LKPTPEGDLEIL	57	[2]	1324.549	−0.233
LPQYL	60.8 *	[1]	632.7653	0.240
FLQP	65.3	[2]	503.5987	0.375
IPGDPGPPGPPGP	65.4	[2]	1154.288	−0.908
WV	65.7	[2]	303.361	1.650
APF	65.8	[3]	333.3873	1.000
MPLW	67.3 *	[1]	545.7032	0.800
TQMVDEEIMEKFR	68.8	[4]	1655.914	−0.869
NH	69	[2]	269.2603	−3.350
IPM	69.5	[5]	359.49	1.600
QPG	70.9	[6]	300.3147	−1.833
QPF	71.7	[6]	390.4393	−0.767
VL	74	[2]	230.3073	4.000
KPA	74.5	[3]	314.3849	−1.233
PFP	74.5 *	[1]	359.4251	−0.133

PQSVLS	74.5 *	[1]	629.7112	0.217
YVPEPF	74.5 *	[1]	750.8493	−0.167
LPGERGRPGAPGP	76.8	[2]	1260.419	−1.146
CAYQWQRPVDRIR	78	[2]	1690.95	−1.138
SPQ	78.9	[6]	330.3409	−1.967
QPQ	79.8	[6]	371.3935	−2.867
GPVGPAGNPGANGLN	81.3	[2]	1291.397	−0.380
GPVGXAGPPGK	83.3	[2]	933.0833	−0.536
TW	84	[2]	305.3336	−0.800
NLGIILR	86.3	[4]	797.9966	1.171
LPVP	87 *	[1]	424.5463	1.200
IA	88	[2]	202.2535	3.150
GPKGDRGLPGPPGRDGM	89.6	[2]	1663.889	−1.353
ML	91	[2]	262.3733	2.850
WA	92.6	[2]	275.3073	0.450
MM	93	[2]	280.4124	1.900
MPVQA	93.3	[7]	544.6727	0.560
GPVGPSGPXGK	93.7	[2]	949.0833	−0.773
FA	94	[2]	236.2706	2.300
PACGGFWISGRPG	96.4	[2]	1304.495	0.046
EMPFPK	97.6 *	[1]	747.9135	−0.983
LPLP	101.0 *	[1]	438.5675	1.100
SPGSSGPQGFTG	101.6	[2]	1078.104	−0.717
LQAFEPLR	103.5	[8]	973.1402	−0.113
LPIIDI	105.4	[9]	682.8584	2.033
IAVPTGVA	106	[10]	726.8783	1.725
GPFPIIV	108.0 *	[1]	741.9373	1.771
HPIK	108.0 *	[1]	493.6068	−1.050
LPYPY	108.3	[2]	651.7683	−0.400
VPITPTL	110	[2]	739.9104	1.129
PGVGGPLGPIGPCYE	116.1	[2]	1412.63	0.120
APR	119.7	[3]	342.3982	−1.433
APFPE	120 *	[1]	559.6195	−0.420
APFPEVF	120 *	[1]	805.9287	0.700
WPW	120.1	[3]	487.5585	−1.133
WQ	120.3	[2]	332.3592	−2.200
YPVEPF	124.7	[7]	750.8493	−0.167
VPITPT	130	[2]	626.7509	0.683
WPI	133	[3]	414.5046	0.667
WI	138.7	[2]	317.388	1.800
LAPSTM	140.8	[11]	618.7563	0.733
WLAHKALCSEKLDQ	141	[2]	1641.912	−0.407
IPAVFK	143	[2]	673.8603	1.300
HL	143.2	[2]	268.3159	0.300
PPGPTGPRGQPGNIGF	146.7	[2]	1548.722	−0.931
WN	148.5	[2]	318.3324	−2.200
MAGVDHI	153.4	[2]	741.8665	0.757
KL	159.8	[12]	259.3489	−0.050
WPF	159.8	[3]	448.5218	0.100
LPQNIPP	160	[2]	777.9283	−0.500

GPFPILV	163.7	[2]	741.9373	1.671
LAHKALCSEKL	165	[2]	1212.488	0.200
TKCEVFRE	166	[2]	1011.167	−0.825
VA	168.2	[2]	188.2266	3.000
FAGDDAPR	168.7	[11]	847.8829	−0.888
VAGTWY	174	[2]	695.7729	0.450
YPG	174	[13]	335.3599	−1.100
IPW	175.3	[3]	414.5047	0.667
LLQLEAIR	177.8	[7]	955.1753	0.775
LCSEKLDQ	186	[2]	935.0733	−0.638
IPAVFKIDA	191	[2]	973.1893	1.178
LKALPMH	193	[2]	809.0493	0.371
YPYY	194.4	[2]	604.6598	−1.375
LPQNIPPLT	205.2	[5]	992.1953	−0.044
WSG	209.9	[14]	348.3587	−0.700
SPVVPF	214.1	[7]	644.7686	1.200
WWW	216	[2]	576.6551	−0.900
VPGEIVE	224.5	[5]	741.8396	0.557
GPMGPXGVK	226.9	[2]	839.0323	−0.422
LTFPGSAED	228	[10]	935.9862	−0.233
APGPAGP	229.1	[9]	565.6269	−0.286
WIQP	237.3	[2]	542.6355	−0.375
WM	243.1	[2]	335.4271	0.500
YPY	243.7	[2]	441.4838	−1.400
GPAV	245.6	[15]	342.3953	1.000
FPF	247	[3]	409.4851	1.333
FPGPIPN	260	[2]	740.8573	−0.200
ILDKVGINY	263	[2]	1034.231	0.489
FSD	275.1	[14]	367.3585	−0.500
WY	281	[2]	367.4045	−1.100
WLAHKAL	286	[2]	838.0205	0.457
GPAGPpGVpGL #	318.1	[2]	918.0693	0.164
TPEVDDEALEK	319.5	[2]	1245.306	−1.264
LPLPL	325	[2]	551.7271	1.640
IQKVAGTW	329	[2]	902.0615	0.138
IVQNNDSTEYGLF	337	[2]	1499.597	−0.415
FL	339.6	[2]	278.3513	3.300
ILDKEGIDY	347.8	[7]	1065.197	−0.367
WRE	350	[2]	489.5316	−2.967
MHQPPQPL	350.4	[2]	947.1262	−1.163
YPL	364.6	[13]	391.4673	0.300
LPLPLL	371.5	[5]	664.8865	2.000
WRD	376	[2]	475.5047	−2.967
VP	380.3	[5]	214.2645	1.300
PPL	390.1	[2]	325.4081	0.200
FAGDDAPRA	393.3	[11]	918.9617	−0.589
WRN	403	[2]	474.52	−2.967
WRK	406	[2]	488.5903	−3.100
IPSK	406.8	[5]	443.5483	−0.450
IP	410	[2]	228.2914	1.450

WRF	413	[2]	507.5927	−0.867
WC	420	[2]	307.3733	0.800
VLVLDTDYK	424.4	[2]	1065.232	0.344
IPPL	428.9	[5]	438.5733	1.275
HPINHR	452.2	[2]	772.8652	−1.917
LA	454	[2]	202.2535	2.800
IPPLTQT	465.1	[5]	768.9173	0.029
WRG	473	[2]	417.4681	−1.933
WT	482.1	[2]	305.3336	−0.800
WRS	483	[2]	447.4943	−2.067
LKGYGGVSLPE	486	[2]	1119.293	−0.045
WRW	487	[2]	546.6294	−2.100
WRT	526	[2]	461.5212	−2.033
FF	546.8	[15]	312.3684	2.800
WW	554.8	[2]	390.4418	−0.900
LPP	563.3	[5]	325.4081	0.200
WRR	570	[2]	516.6037	−3.300
VLGP	580.4	[2]	384.476	1.500
WRY	640	[2]	523.5921	−2.233
WS	643.5	[2]	291.3067	−0.850
RP	657.2	[5]	271.3195	−3.050
YP	658.1	[2]	278.3078	−1.450
WRH	670	[2]	497.5573	−2.867
YPFPGPIPN	670	[2]	1001.15	−0.478
WRM	673	[2]	491.6147	−1.167
SPTVMFPPQSVL	676.3	[2]	1302.558	0.525
WRA	690	[2]	431.4949	−1.200
WRQ	720	[2]	488.5469	−2.967
ILELA	721.1	[7]	557.6933	2.080
WRI	730	[2]	473.5756	−0.300
YPFPGP	749.2	[5]	676.7699	−0.617
WRP	780	[2]	457.5328	−2.333
VR	826.1	[2]	273.3354	−0.150
MP	870	[2]	246.3304	0.150
AL	882.1	[2]	202.2534	2.800
HP	902.8	[5]	252.2731	−2.400
WRL	903	[2]	473.5756	−0.533
RRDY	930	[16]	608.655	−3.450
LW	993.4	[2]	317.388	1.450
PGPIHNS	1000	[2]	720.7833	−0.943
QPLPPT	1013.8	[5]	651.7606	−0.867
KVEPLP	1071.9	[17]	681.8304	−0.433
LQP	1181.1	[2]	356.4222	−0.433
RL	1200	[16]	287.3623	−0.350
IPPLTQTPV	1300	[2]	965.1693	0.311
TPVVVPP	1408.9	[5]	707.8682	1.014
PQNIPPL	1500	[2]	777.919	−0.500
DPF	1540	[16]	377.3971	−0.767
GF	1547.1	[18]	222.2438	1.200
GPGSPGGPL	1638.3	[2]	737.8173	−0.378

MW	1691.4	[2]	335.4271	0.500
QPHQPLPPT	1754.8	[5]	1014.149	−1.500
LR	2083.6	[12]	287.3623	−0.350
MGSF	2120	[16]	440.5206	0.875
LP	2370	[2]	228.2914	1.100
TP	2370	[2]	216.2369	−1.150
VPPFIQPE	2500	[2]	926.0803	−0.038
SL	2517.1	[2]	218.2529	1.500
KP	2540	[2]	243.3061	−2.750
GL	2615	[2]	188.2267	1.700
GPA	2870	[16]	243.2627	−0.067
PAL	2943.1	[17]	299.3702	1.333
EK	3216.7	[2]	275.3049	−3.700
FP	3630	[2]	262.3085	0.600
IHF	3770	[16]	415.4925	1.367
KRIHF	4110	[16]	699.8543	−0.860
PP	4343.5	[18]	212.2486	−1.600
VC	5413.4	[15]	220.2926	3.350
SP	5980	[2]	202.2101	−1.200
KA	6270	[2]	217.2682	−1.050
AAATP	6470	[2]	429.4733	0.620
AP	7950	[2]	186.2106	0.100
AAAAG	8130	[2]	359.3824	1.360
GGPAGPAV	8139.1	[18]	624.6948	0.425
AA	9400	[2]	160.1728	1.800
GP	9690	[2]	172.1839	−1.000

* Estimated based on their reported percent relative potency.
# p, hydroxyproline

References

1. Boots, J.W.P. Protein hydrolysate enriched in peptides inhibiting DPP-IV and their use. United States Patent US 2009/0075904 A1.
2. Lacroix, I.M.E.; Li-Chan, E.C.Y. Food-derived dipeptidyl-peptidase IV inhibitors as a potential approach for glycemic regulation—Current knowledge and future research considerations. *Trends Food Sci. Technol.* **2016**, *54*, 1–16.
3. Nongonierma, A.B.; Dellafiora, L.; Paolella, S.; Galaverna, G.; Cozzini, P.; FitzGerald, R.J. In Silico Approaches Applied to the Study of Peptide Analogs of Ile-Pro-Ile in Relation to Their Dipeptidyl Peptidase IV Inhibitory Properties. *Front Endocrinol.* **2018**, *9*, 329.
4. Song, J.J.; Wang, Q.; Du, M.; Ji, X.M.; Mao, X.Y. Identification of dipeptidyl peptidase-IV inhibitory peptides from mare whey protein hydrolysates. *J. Dairy Sci* **2017**, *100*, 6885–6894.
5. Nongonierma, A.B.; FitzGerald, R.J. Structure activity relationship modelling of milk protein-derived peptides with dipeptidyl peptidase IV (DPP-IV) inhibitory activity. *Peptides* **2016**, *79*, 1–7.
6. Taga, Y.; Hayashida, O.; Kusubata, M.; Ogawa-Goto, K.; Hattori, S. Production of a novel wheat gluten hydrolysate containing dipeptidyl peptidase-IV inhibitory tripeptides using ginger protease. *Biosci. Biotechnol. Biochem.* **2017**, *81*, 1823–1828.
7. Nongonierma, A.B.; Paolella, S.; Mudgil, P.; Maqsood, S.; FitzGerald, R.J. Identification of novel dipeptidyl peptidase IV (DPP-IV) inhibitory peptides in camel milk protein hydrolysates. *Food Chem.* **2018**, *244*, 340–348.
8. Wang, F.; Yu, G.; Zhang, Y.; Zhang, B.; Fan, J. Dipeptidyl Peptidase IV Inhibitory Peptides Derived from Oat (Avena sativa L.), Buckwheat (Fagopyrum esculentum), and Highland Barley (Hordeum vulgare trifurcatum (L.) Trofim) Proteins. *J. Agric. Food Chem.* **2015**, *63*, 9543–9549.

Commented [M1]: Please provide the website and accessed date (Date Month Year)

9. Zhang, Y.; Chen, R.; Chen, X.; Zeng, Z.; Ma, H.; Chen, S. Dipeptidyl Peptidase IV-Inhibitory Peptides Derived from Silver Carp (Hypophthalmichthys molitrix Val.) Proteins. *J. Agric. Food Chem.* **2016**, *64*, 831–839.
10. Lammi, C.; Zanoni, C.; Arnoldi, A.; Vistoli, G. Peptides Derived from Soy and Lupin Protein as Dipeptidyl-Peptidase IV Inhibitors: In Vitro Biochemical Screening and in Silico Molecular Modeling Study. *J. Agric. Food Chem.* **2016**, *64*, 9601–9606.
11. Liu, R.; Zhou, L.; Zhang, Y.; Sheng, N.J.; Wang, Z.K.; Wu, T.Z.; Wang, X.Z.; Wu, H. Rapid Identification of Dipeptidyl Peptidase-IV (DPP-IV) Inhibitory Peptides from Ruditapes philippinarum Hydrolysate. *Molecules* **2017,** 22.
12. Sato, K.; Miyasaka, S.; Tsuji, A.; Tachi, H. Isolation and characterization of peptides with dipeptidyl peptidase IV (DPPIV) inhibitory activity from natto using DPPIV from Aspergillus oryzae. *Food Chem.* **2018**, *261*, 51–56.
13. Ibrahim, M.A.; Serem, J.C.; Bester, M.J.; Neitz, A.W.; Gaspar, A.R.M. Multiple antidiabetic effects of three alpha-glucosidase inhibitory peptides, PFP, YPL and YPG: Dipeptidyl peptidase-IV inhibition, suppression of lipid accumulation in differentiated 3T3-L1 adipocytes and scavenging activity on methylglyoxal. *Int. J. Biol. Macromol.* **2018**, *122*, 104–114.
14. Sila, A.; Alvarez, O.M.; Haddar, A.; Frikha, F.; Dhulster, P.; Nedjar-Arroume, N.; Bougatef, A. Purification, identification and structural modelling of DPP-IV inhibiting peptides from barbel protein hydrolysate. *J. Chromatogr. B Analyt. Technol. Biomed. Life Sci.* **2016**, *1008*, 260–269.
15. Neves, A.C.; Harnedy, P.A.; O'Keeffe, M.B.; FitzGerald, R.J. Bioactive peptides from Atlantic salmon (Salmo salar) with angiotensin converting enzyme and dipeptidyl peptidase IV inhibitory, and antioxidant activities. *Food Chem.* **2017**, *218*, 396–405.
16. Lin, Y.S.; Han, C.H.; Lin, S.Y.; Hou, W.C. Synthesized Peptides from Yam Dioscorin Hydrolysis in Silico Exhibit Dipeptidyl Peptidase-IV Inhibitory Activities and Oral Glucose Tolerance Improvements in Normal Mice. *J. Agric. Food Chem.* **2016**, *64*, 6451–6458.
17. Ji, W.; Zhang, C.; Ji, H. Two Novel Bioactive Peptides from Antarctic Krill with Dual Angiotensin Converting Enzyme and Dipeptidyl Peptidase IV Inhibitory Activities. *J. Food Sci.* **2017**, *82*, 1742–1749.
18. Neves, A.C.; Harnedy, P.A.; O'Keeffe, M.B.; Alashi, M.A.; Aluko, R.E.; FitzGerald, R.J. Peptide identification in a salmon gelatin hydrolysate with antihypertensive, dipeptidyl peptidase IV inhibitory and antioxidant activities. *Food Res. Int.* **2017**, *100*, 112–120.
